# “Empowering” Cardiac Cells via Stem Cell Derived Mitochondrial Transplantation- Does Age Matter?

**DOI:** 10.3390/ijms22041824

**Published:** 2021-02-12

**Authors:** Matthias Mietsch, Rabea Hinkel

**Affiliations:** 1 Laboratory Animal Science Unit, Leibniz-Institut für Primatenforschung, Deutsches Primatenzentrum GmbH, Kellnerweg 4, 37077 Göttingen, Germany; mmietsch@dpz.eu; 2 DZHK (German Center for Cardiovascular Research), partner site Göttingen, 37077 Göttingen, Germany

**Keywords:** stem cell, aging, senescence, mitochondria, transplantation, cardiovascular, heart

## Abstract

With cardiovascular diseases affecting millions of patients, new treatment strategies are urgently needed. The use of stem cell based approaches has been investigated during the last decades and promising effects have been achieved. However, the beneficial effect of stem cells has been found to being partly due to paracrine functions by alterations of their microenvironment and so an interesting field of research, the “stem- less” approaches has emerged over the last years using or altering the microenvironment, for example, via deletion of senescent cells, application of micro RNAs or by modifying the cellular energy metabolism via targeting mitochondria. Using autologous muscle-derived mitochondria for transplantations into the affected tissues has resulted in promising reports of improvements of cardiac functions in vitro and in vivo. However, since the targeted treatment group represents mainly elderly or otherwise sick patients, it is unclear whether and to what extent autologous mitochondria would exert their beneficial effects in these cases. Stem cells might represent better sources for mitochondria and could enhance the effect of mitochondrial transplantations. Therefore in this review we aim to provide an overview on aging effects of stem cells and mitochondria which might be important for mitochondrial transplantation and to give an overview on the current state in this field together with considerations worthwhile for further investigations.

## 1. Introduction

With 600,000 deaths in the United States and 18 million overall, cardiovascular diseases represent the primary cause of death worldwide [1,2]. In addition to their enormous health, ethical and financial issues, they are expected to increase in the coming years especially due to the rise of aging individuals worldwide, their increasingly extending life span and the occurrence of concomitant age-related cardiovascular changes [3]. One reason for the disturbing high mortality rate is the circumstance that cardiac tissue is not able to regenerate diseased cells properly since the yearly tissue turnover rate is only around 1%. Insults like ischemia caused by myocardial infarction and subsequent reperfusion or cardiomyopathies therefore lead to substantial impairments of cardiac function. Traditional treatments with pharmaceutical and exogenous substrate interventions (statins, beta-adrenergic blockers, angiotensin converting enzyme inhibitors, aspirin, clopidogrel etc.) and/or surgeries provide often only limited success with a remaining negative impact on post ischemic recovery and cell viability [4]. Up to date reliable treatments against cardiac damage and for cardiac rejuvenation are urgently needed and under extensive investigation [5].

In the past decades the field of stem cell based treatment strategies emerged as a promising approach to prevent or tackle consequences of disease-caused cardiovascular changes in various application areas. By aiming to rescue damaged cells and regenerate damaged tissue or modulate inflammatory pathways extensive research has been conducted over the last decades mainly with mixed or negative results. Hurdles like the optimal application strategy, poor survival and engraftment of the cells as well as a lack of proliferation and the danger of rejection as well as the need for immunosuppressive agents presented drawbacks in the use of cell-based treatment approaches [6,7,8,9]. In addition recent research implicated paracrine factors of the stem cells and the microenvironment to enhance the benefit compared to direct stem cell applications used [10,11,12,13]. Based on these findings so called “stem-less” approaches emerged as interesting alternatives to the use of stem cells in cardiac medicine. Various positive effects have been described and were attributed to the beneficial effects such as improvement of the microenvironment, the removal of senescent cells, the optimization and application of exosomes, microRNAs (miRNAs) and latest also mitochondria [14,15,16]. Especially the latter might represent an interesting therapeutic target: Cardiomyocytes heavily rely on an adequate energy metabolism and mitochondria constitute the main energy source of cells. Mitochondrial changes have been repeatedly found in cardiovascular alterations and targeting them yielded improvements in function and regeneration. In recent years mitochondrial transplantation therefore emerged as an interesting and so far, promising therapeutic option in a variety of diseases, including cardiovascular.

However despite the reported positive effects, previous in vivo studies used mainly autologous derived mitochondria. With regard to the probable patient cohort- old and diseased individuals- and the observable aging effects of various cell types and mitochondria themselves this might pose obstacles in the application of this approach. The questions arise if mitochondria from somatic tissues of the same individual are safe, effective and expedient in these cases or if other cell sources like, for example, stem cells or mitochondria derived from younger individuals would exert a more beneficial effect.

This review therefore aims to address possible aspects important for the consideration of aged stem cells as sources for mitochondrial transplantation and to provide a current overview over the recent in vivo research involving this new therapeutic option. Lastly considerations for further research in this promising area are given.

## 2. Aging Effects on Stem Cells

Aging represents a decline in functions on the body/organismal level but also on the cellular level. This section aims to summarize hallmarks of stem cell aging which might be important for their consideration as therapeutical mitochondria sources.

As all cells, also stem cells show signs of aging: Although the hematopoietic stem cell (HSC) pool expands over the lifespan, cells exhibit reduced repopulation and self-renewal capacity, increased clonality towards a more myeloid bias, altered migratory capacities, telomere shortening, the release of cytokines and epigenetic dysregulations [17,18,19,20,21,22,23,24,25]. Different mechanisms of aging like replicative aging, chronological aging and exhaustion play a part in the senescent phenotype [26]. 

Associated diseases like anemia, adaptive immune compromise (possibly due to loss of lymphoid cell numbers) and malignancy are age-associated [27]. Molecular mechanisms of stem cell aging include increasing DNA damage, cellular senescence, increased production of reactive oxygen species (ROS) and mitochondrial dysfunction. 

In addition, stem cells show transitioning to a senescent phenotype by increasing senescent markers (p53 and p16) and reducing stem cell markers like c-kit, Nanog, Oct4 and KLF4 [28]. The decline in regenerative potential might be due to an increasing inability of, for example, cardiac progenitor cells (CPCs) to respond to signaling [25]. The exosomes of aged mesenchymal stem cells (MSCs) exhibit different profiles than exosomes derived from younger cells [29], whereas extracellular vesicles (EVs) from aged HSCs showed no decline in respiring mitochondria [30].

Stem cells are typically long living and a subpopulation remains in a dormant quiescent state for a long period of time [31]. These dormant cells are characterized by minimal basal metabolic activity, low mitochondria content and a reliance on glycolysis for energy production, which might result in a reduced ROS production in these cells [32]. Depending on the tissue stem cells were found to switch between the dormant and active state. The quiescent state seems to minimize replicative stress and DNA damage accumulation [33]. The activation of stem cells leads to DNA damage [34] possibly due to their sensitivity to damage during cell divisions [35]. This was supported by Wilson et al. who showed that the most potent repopulation active HSCs are the ones undergoing the least divisions [36,37].

In contrast to these findings, Beerman and colleagues showed that especially dormant cells accumulate strand breaks (possibly due to less reparative mechanisms in the quiescent state) so it seems that neither extreme dormancy nor extreme proliferation are optimal criteria to identify HSCs with the least genomic damage [38]. Supporting this thesis, Bernitz et al. described a “cellular memory” of stem cells and elaborated that long-term regenerative potential might be lost upon the fifth division [39].

Normally, not all stem cells are dormant and just a fraction of stem cells enters quiescence. These cells seem to be able to retain their proliferative and regenerative potential despite other cells acquiring a senescent phenotype. However, with aging, for example, stem cells of muscle tissues (satellite cells) seem to undergo a switch from quiescence to senescence thereby loosing self-renewal and regenerative capacities [40,41].

Taken together, the divisional history of hematopoietic stem cells might affect their genomic stability and likely be a key factor when selecting the most potent stem cells for therapeutic usage like mitochondrial transplantations.

## 3. Heterogeneity in Stem Cell Aging

Although there is a global decline in cellular functions with aging, not all individuals and cells age with the same speed and inter individual as well as intra individual variations can be observed. When monitoring, for example, mice there are individual differences regarding mitochondrial functions and longevity throughout their lives [42]. Although traditionally being thought to age in a similar fashion, during recent years, evidence of a certain heterogeneity in stem cell aging became apparent indicating that stem cells might differ in regard to their biological age compared to the same chronological age [27]. Besides the already mentioned heterogeneity in replicative history, this section summarizes studies investigating different aspects of stem cell aging implicating the occurrence of a certain heterogeneity on different levels which might be interesting for therapeutical approaches.

Although HSCs in general show a decline in autophagy (regulating cellular health), regeneration potential and self-renewal activity with aging [43], a subset of cells maintains its autophagy abilities and regenerative capacity [44]. In addition, differences depending on the stem cell source became apparent: Intestinal stem cells show an increase in numbers but a decrease in proliferative capacity [45,46]. In the muscle, stem cells decline together with a decrease in regenerative capacity [47,48]. In the central nervous system a decline in function and regenerative capacity is also seen for the stem cells [49] whereas epidermal stem cell numbers remain stable throughout aging [50].

Kirschner et al. showed a heterogeneity in stem cell aging by investigating murine HSCs with single cell transcriptomic analyzes. They revealed the existence of a subset of bone marrow derived stem cells in old mice which displayed an upregulated p53 pathway and JAK/STAT gene signatures, a decrease in proliferation and a myeloid bias, whereas other stem cells did not show these alterations. The authors proposed that a continuously/prolonged proliferation induced by an activated JAK/STAT pathway of HSCs might lead to their exhaustion in terms of p53 upregulation, decline in proliferative capacity and myeloid bias [51].

As described above, in the skeletal muscle studies implicate that satellite cells seem to decline with age and to display reduced proliferative capacities [47,52,53,54]. However other studies found differing results implicating a stable satellite cell pool [55]. These contradicting findings might be due to the heterogeneity of this population with a subset (in contrast to the majority of satellite cells) seemingly surviving aging effects [56].

Possible explanations for these heterogenic stem cell aging are differences in cell divisions affecting the mitochondria pool of the cells, individual varying regenerative capacities or cell cycle rates. When stem cells divide there is an asymmetric cell division which leads to two different daughter cells. Daughter cells with retained aged mitochondria during division differentiate, daughter cells with young mitochondria maintain stem cell like properties. This asymmetric apportioning of mitochondria seems to preserve stemness properties [57] and might be an important factor for the occurrence of heterogeneity.

Just recently Stumpf et al. provided an interesting and exciting model theoretically illustrating the rise of heterogeneity in aging proliferating stem cell populations [35]. The model describes the existence of a heterogeneous mix of cells with different innate regenerative abilities. They discussed heterogeneity in the context that a seemingly homogeneous cell population may have a similar cell type but many functional cell types where individual cells may vary regarding their molecular status and therefore differ in cell states. Studies found, for example, differences in cell cycle times/rates of HSCs thereby presenting a lack of synchronization. Regarding aging, this would mean that these asynchronies potentiate and that in the old organism there is a variability of different cells in the stem cell pool with a heterogeneity in regard to mitotic history [35]. If this variety is of importance for cell function is still questionable since numerous studies found alterations of cell and mitochondrial functions to appear only above a certain threshold of (accumulated) mutations. Regarding models of heterogeneity, another interesting model was proposed earlier by Glauche et al. also describing theoretically the potential generation of heterogeneous cell populations [58].

A further explanation for intercellular variances was provided by the group of Lauridson and colleagues: Using Single-cell RNA sequencing (RNA-seq) they showed that differences in cell cycle activity constituted the main driver of transcriptional heterogeneity in HSCs [59].

Considering these cell individual differences, it might be of importance to find phenotypic markers for the identification of the different stem cell subsets: Exploring markers for heterogeneity showed that c-kit low HSCs are more quiescent and display delayed multi lineage differentiation (c-kit low: enhanced self-renewal and long-term repopulating potential). Other markers like CD34 or CD150 for lymphoid and myeloid bias, respectively, might be also of interest for the identification of suitable stem cell sources [33].

Not just the cells within a specific tissue type seem to differ in various phenotypes but there are also variations between different tissues. Identifying an optimal tissue as (stem) cell source for therapeutic applications is important since, for example, bone marrow (BM) derived stem cells consist of a mix of cell populations including MSCs, HSCs, embryonic-like stem cells and mononuclear (MN) stem cells [60]. In direct comparison, cortical bone stem cells seem to be more effective than cardiac derived and MSCs showing higher proliferative capacity as well as better survival and immunomodulatory capacity (lower levels of IL-1α, secreted phosphoprotein-1 and IL-18) [61]. Functional comparisons of BM MN cells, MSCs, skeletal myoblasts and fibroblasts in mice revealed BM MN cells to show an enhanced survival rate [62]. In contrast to these reports, by directly comparing BM-MSCs, human cardiosphere-derived cells (CDCs), MSCs from adipose tissue and BM-MN cells, Li and colleagues found CDCs to exhibit the highest secretion of angiogenic and anti-apoptotic factors and to produce the best improvement of cardiac function [63].

These studies prove the existence of various levels of heterogeneity in stem cells (ranging from the genomic level over their phenotypes and functional properties) and their aging process which might be of importance for further investigations when optimal cell sources for therapeutical interventions might be of interest.

## 4. Mitochondria

Mitochondria, often called the power house of the cell, not only provide a constant and vital supply of ATP and NADH, but extensive research also revealed far more functions like controlling of apoptosis and participation in biosynthesis [27]. They furthermore act as signaling organelles involved in homeostasis of calcium and inflammation, forming a network of fission and fusion in the cell. Their generated ROS act as signal molecules. In addition, mitochondria might act as biomarkers for certain diseases and prognostic factors.

Mitochondria are located in abundance in cardiomyocytes. They constitute around 30% of volume in cardiac muscle cells [64]. As the main source for energy supply, mitochondria play an essential role in cell metabolism and cardiac function. In heart diseases not only processes like structural remodeling and oxidative stress but also mitochondrial dysfunction contributes to the development of heart failure [65,66,67]. Consequently, changes in mitochondria morphology have been found in cardiovascular diseases [64,68]. Mitochondria therefore seem to be a promising therapeutic substance for the treatment of these diseases: When cells are damaged (e.g., by ischemia), ATP production usually conducted by mitochondria declines and results in cell damage or cell death. The released mitochondrial components like mitochondrial DNA (mtDNA) might present damage associated molecular patterns (DAMPs) which lead to pro inflammatory immune responses contributing to the inflammatory phenotype (SASP) associated with senescence [69]. An accumulation of mitochondrial damage has been observed during ageing and in reperfusion following cardiac ischemia the mitochondria-generated ROS cause further damage [70]. Restoration of cardiac function and energy homeostasis is therefore one promising approach in treating cardiac diseases. Preserving myocardial energetics by targeting mitochondria might be one possible way to achieve this [71]. However, modulation of the existing (damaged) mitochondria seems to be unsuccessful.

Since they are maternally inherited and of bacterial origin, mitochondria possess their own, from the nuclear DNA distant genetic code, the circular mtDNA. However, their protein metabolism is closely linked to the cell since the majority of mitochondrial proteins is at least partially encoded in nuclear DNA. mtDNA alone only encodes for 13 protein subunits. In cells, mutated mtDNA is often found in proximity to wild type, non-mutated mtDNA. This phenomenon called heteroplasmy will be discussed as part of the following section.

## 5. Mitochondrial Aging

**Figure 1:**
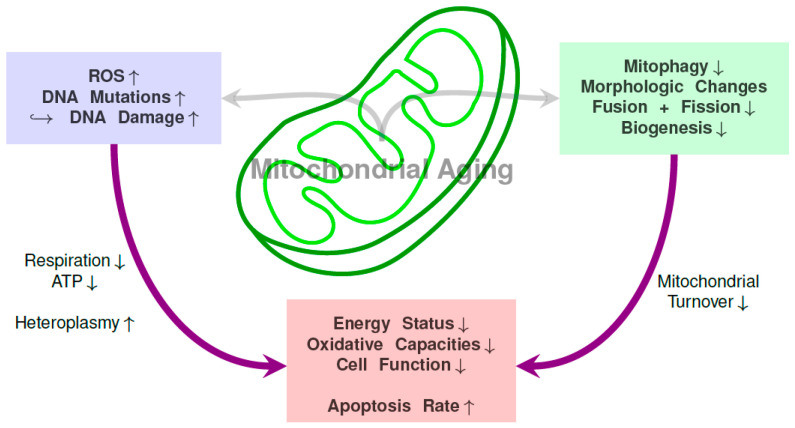
Overview over connections between mitochondrial DNA mutations, functional mitochondrial changes and resulting consequences on the mitochondria and cell level.

Together with the age-related decline in (stem) cell function, mitochondria also display signs of senescence: They show a decrease in mitochondrial volume density, a decline in activity of mitochondrial enzymes like cytochrome C oxidase and citrate synthase, a decrease in respiratory rates and ATP production, cardiolipin peroxidation and an increase in ROS production [72,73,74]. Mitophagy rates decrease during aging [75,76] and there are alterations in the mitochondrial integrity [77]. The changes of cardiolipin content are not clear since some studies suggest a decline while others describe stable conditions. Figure 1 gives a general overview over mitochondrial changes associated with aging.

Traditionally it has been thought that the increased ROS occurrence at the mitochondrial membrane and its near localization to the mtDNA are the main cause for the observable increasing mtDNA damages and important for their functional decline. However, according to extensive research conducted during the last decades this theory of ROS damage might be not the critical route for mitochondrial aging. Instead, the accumulation of molecular alterations of mtDNA with age might be the driving mechanism [78]. These molecular DNA alterations are associated with the occurrence of diseases [79]. Spontaneous errors during DNA replications may occur and are responsible for a majority of mutations in mtDNA. mtDNA displays a much higher mutation rate than nuclear DNA probably at least partly due to ROS-induced damages, a lack of histone-like protection and low repair mechanisms. Recent studies showed the accumulation of spontaneous mutations to likely be the main reason for these increases [80]. Point mutations and deletions represent the most common alterations in mtDNA. In stem cells heteroplasmy, the occurrence of mutated and wildtype mtDNA in a single cell is an observable phenomenon: Mutation rates of mtDNA seem to display tissue specific variations with post mitotic tissues like heart and muscle showing higher heteroplasmy whereas mutations in skin and blood appear to be lower and of lesser impact [81]. In direct comparison different tissues display varying rates of mitochondrial mutations (e.g., mutations of mtDNA4977 is higher in skeletal muscle compared to heart and kidney; lower 3243 A→G mutations in skeletal muscle compared to kidney) associated with different metabolic and senescent characteristics [82] with heteroplasmy (hypervariable regions of the noncoding region) in skeletal muscle being the highest (compared to blood, heart and brain) [83]. In direct comparison between somatic tissues and stem cells, somatic cells display higher mutation rates than stem cells: when liver tissue was investigated, Brazhnik and colleagues found around twofold higher somatic mutation frequencies of differentiated hepatocytes compared to liver stem cells. In addition, an increase of single-nucleotide variants with age and three times more base substitution mutations in old cells versus young cells were seen [84].

In cardiovascular diseases the occurrence of heteroplasmy might be a possible rescue mechanism for the heart: by combining, for example, ischemia-damaged mitochondria with new mitochondria supplied by mitochondrial transplantation heteroplasmy via constant fusion and fission may lead to a dilution effect of these damages and thereby maybe rescuing the old mitochondria. However, this possible rescue mechanism is not endless: exceeding a certain threshold of mitochondrial mutations they are associated with the occurrence of diseases [85]. The increasing occurrence of mutations might lead to a higher incidence of tumorigenicity [86] and, for example, in muscle-derived stem cells functional decline with advancing age due to dysfunctional autophagy has been observed [87]. On the cellular level disruption of mitochondrial function can occur after exceeding the threshold [80]. In this context, the role of important enzymes like Pol y, PolG2, Twinkle, TFAM, MGME1 and RNase H1 has been extensively described by DeBalsi et al. [80]. 

As a side note mtDNA mutations can also be seen in induced pluripotent stem cells (iPSCs): These cells, which are reprogrammed from somatic cells, show also age-related alterations like a rise of mtDNA mutations [81], which could impact respiratory function. La Sardo et al. observed increased DNA methylation in old donor PBMCs which was mirrored in the corresponding iPSCs. Aged iPSCs seemed to display a resistance to demethylation during reprogramming and a slight demethylation deficiency in an amount of approximately 5% [86]. The authors of the same study could show that most of the aberrant DNA methylation could be erased with extended passaging of the iPSCs. 

Taken together, mutations of mtDNA and mutations of mitochondrial enzymes can lead to heterogeneity of cell populations during aging [88]. As a consequence, mutations of mtDNA vary both between body tissues, between cells of the same tissue and even within single cells [89,90]. Therefore a thorough screening for mutations and assessment of metabolic status should be conducted when stem cells or iPSCs from old donors are intended for clinical use.

Concomitant with these findings, it is not surprising that tissues with high energy demand and mitochondrial activity like brain and muscle are prone for the accumulation of mtDNA mutations. As tissues are differently active, differences in the occurrence of mitochondrial mutations and, as a consequence, mitochondrial diseases have been observed. Tissues with high mitochondrial respiration like skeletal muscles are more affected than other organs [91]. By directly comparing heart, skeletal muscle, liver, kidney, brain and brown fat tissue of mice, Herbers et al. found higher levels of mtDNA damage in tissues with higher mitochondrial respiration (heart, skeletal muscle, brown fat) but at the same time signs of mtDNA recombination and strand-coupled replication in these tissues. In contrast, liver and kidneys showed the least mtDNA damage and no signs of mtDNA recombination and they might use strand-asynchronous mechanism for replications. The authors postulated that the occurrence of mtDNA recombination might be a repair mechanism for mtDNA damage in post mitotic cells. These differences might account for the tissue specific differences in mtDNA disorders mostly affecting muscle and brain which might possibly be due to a failure to maintain genome integrity [79].

Besides the genomic level, Mansell et al. recently investigated mitochondrial aging phenotypes from mice showing that aging BM-HSCs display a decrease in mitochondrial membrane potential in accordance with a lower transcriptional rate and associated changes of ROS. Despite this, a fraction of these stem cells mimicked characteristics (increased mitochondrial membrane potential and transcription rate) of young mice. They further showed that with serial injections of mitoquinol a partially rejuvenation in terms of improvements of mitochondrial membrane potential and transcription rate and a decrease in intracellular ROS could be seen [27]. Therefore in addition to genetic and proteomic markers membrane potential might be useful for differentiation and identification of “young” cells among the “old” HSC pool.

However, various studies showed not only inter-tissue variability but also inter- cellular and/or inter-mitochondria differences/mosaicism within each tissue, for example, in skeletal muscle, aortas and heart [92,93,94]. Cell to cell variability or “mosaicism” increases with age, a phenomenon which has been extensively reviewed by Hahn and colleagues [95]. Cardiac cells for example display two different types of mitochondria—SubSarcolemmal (SS) mitochondria and InterMyoFibrillar (IMF) mitochondria, similar to the skeletal muscle. They show different behaviors during aging with SSM producing greater amounts of ROS and showing higher rates of fragmentation and degradation, while IMF are more susceptible to apoptotic stimuli and mitochondrial permeability transition pore (MPTP) opening [96]. However these two subpopulations have been shown to be interconnected in skeletal muscle questioning the functional relevance of these two different populations. Mitochondria in skeletal muscle affected by sarcopenia, a muscle loss which can be seen during the aging process, display alterations in morphology: SSM and IMF morphologies change with age and an increase in mitochondrial fusion index (Mfn2-to-Drp1 ratio) can be seen [97], which could alter mitochondrial function. In addition old muscles show smaller mitochondria and increased fission proteins [98].

To investigate this intra cell variability in mitochondria, differences and potentials of each mitochondrion have to be monitored. Using a micro-respirometer, Pham et al. were able to reveal differences in single mitochondria and not only presented an elegant method for single mitochondria screenings but also supported the assumption of the existence of a functional heteroplasmy besides the apparent genetic heteroplasmy [99].

Another cause of the mitochondrial decline might be the impaired mitochondrial fission and fusion with age. A functioning homeostasis between fusion and fission is necessary to maintain cellular function [100] and abnormalities cause SC dysfunction. Normally, impairments of mitochondria are counteracted with constant fission, fusion and mitophagy but aging cells display an increasing inability to get rid of damaged mitochondria [69]. Mitochondrial homeostasis between fusion and fission is important to maintain regular cell functions during the lifetime. With aging a shift in mitochondrial dynamics toward fission can be observed in Drosophila ovarian germline stem cells affecting stem cell homeostasis. This is accompanied by an increased occurrence of fragmented mitochondria, loss of membrane potential and a disrupted lipid homeostasis [101]. These basic investigations suggest another functional mitochondrial decline during the aging process which might be important for therapeutic uses.

Interestingly, bringing together the following two independent investigations could enlighten the connection between asymmetric apportioning and functional mitochondrial differences: Twig et al. found in accordance with the aforementioned studies on heterogeneity in stem cells that asymmetric apportioning leads to daughter cells with different mitochondrial membrane potentials [102]. Together with the paper by Mansell and colleagues showing heterogeneity in mitochondrial membrane potentials during aging [27] this could partly explain the observed variances in stem cell senescence and undermines the necessity for a careful screening when stem cells and especially mitochondria are to be used in therapeutical circumstances.

In contrast to aging alterations the effect of diseases like diabetes on mitochondria is less clear [103]: Minet et al. found in vitro impaired mitochondrial functions in type 2 diabetes-derived myotubes due to impaired ATP synthesis [104]. In diabetic rats, mitochondrial ATP content was lower compared to healthy individuals, whereas no obvious functional cardiac differences were seen [105]. In pathological diabetic conditions changes in morphology, superoxide production, cardiolipin content and dysfunctional mitochondrial protein import were seen in IMF of type 1 diabetic mice [106,107,108], whereas in type 2 diabetes the SSM seem to be more affected [109].

## 6. Mitochondrial Transplantation for Heart Diseases

In the context of paracrine functions, cells like MSCs and astrocytes can physiologically transfer different cellular content to other cells via the formation of nanotubes or the release of microvesicles and exosomes [110]. The excretion of these extracellular bodies helps damaged cells to recover, restore metabolic activity or modulate the inflammatory phenotype of immune cells [111,112,113]. A significant part of the positive effects is attributable to the transfer of mitochondria. Mitochondrial transfer/transplantation has emerged as an interesting tool for therapies, for example, in the neurological and cardiovascular field. The replacement or application of additional mitochondria was subject of interest during recent years. In contrast to the much bigger stem cells, with a size of 250–1000nm [114,115,116] the application of mitochondria in the heart poses a reduced risk of occlusion of vessels. Consisting of a lipid double membrane similar to cells, they furthermore pose a reduced risk for immunological reactions compared to genetically different stem cells [117].

Mitochondrial transplantations have been so far successfully used in neurological fields [118,119], liver infarcts, kidney injuries [105], acute lung injury [120], acute limb injury [121] and in various cardiovascular settings. Especially in the field of cardiovascular research, interesting studies have been published during the course of the last years. Based on promising effects of experiments in vitro latest in vivo studies particularly by the group of McCully have been published [4,64,105,114,115,116,120,121,122,123,124,125,126,127,128,129,130,131,132,133]. Table 1 gives an overview of the literature using mitochondrial transplantation for the treatment of cardiovascular diseases.

Excitingly, all of the mentioned studies showed beneficial effects of mitochondrial transplantations both in explanted hearts as well as in vivo in mice, rats, rabbits and especially pigs. Furthermore, as shown below up to date one study describing the first therapeutic application of mitochondrial transplantation in children has been published [124].

Following the first description of mitochondrial transplantation by McCully et al. in 2009, various aspects of this therapeutic option have been investigated and the physiological processes further and further enlightened. McCully and coworkers used autologous derived mitochondria from heart tissue in an ischemia-reperfusion model of the Langendorff perfused rabbit heart and transplanted the mitochondria via direct injection in the affected tissue [4]. A decrease of cardiac biomarkers concomitant with an increase of ATP content in the tissue and an improvement of cardiac function was the result. Transplanted one minute before reperfusion, the mitochondria were viable (<0.05% nonviable) and alive after 120 min of reperfusion. They could be located near the myocytes but not within.

By using a similar approach, Cowan and colleagues could show that not only the direct injection but also the intra coronary application induced beneficial effects on the heart by enhancing regional and global myocardial function [123]. Interestingly, in addition to these experiments, in another subset of the study mitochondria derived from human adult cardiac fibroblasts were used for imaging purposes. For this approach human mitochondria were labeled with 18F-R6G ± iron oxide nanoparticles, injected after ischemia and the hearts were subsequently imaged by PET and micro-CT as well as histological examinations were performed. Transmission electron microscopy showed the mitochondria to be intact and viable (indicated by electron density with cristae morphology). Throughout reperfusion, more than 70% of the 18F-R6G-labeled mitochondria remained within the injected hearts. The staining of the human mitochondria revealed their presence within the interstitial spaces between cardiomyocytes and partially co-localization of injected mitochondria with cardiomyocytes. In fact, 43.52% ± 4.46 of the mitochondria could be associated or were found within cardiomyocytes. In perfused tissue infused mitochondria were associated with blood vessels, cardiomyocytes and the interstitium. 24.76% ± 2.50 and 23.64% ± 2.42 of the mitochondria were colocalized with cardiomyocytes and blood vessels, respectively.

Using pectoralis muscle tissue as a source for mitochondria, Masuzawa et al. proved the application of this concept in living rabbits and followed them for up to 28 days [128]. Mitochondria injected into the myocardium shortly before reperfusion displayed an improved myocardial function, a decrease in infarct size and necrosis (TUNEL^+^ cells and caspase 3 activity), a decrease in cardiac biomarkers for myocardial infarction and an increase in total ATP content in the area at risk. Importantly regarding safety issues no arrhythmogenicity, inflammatory effects (hsCRP, IL6, TNFa) or autoimmune responses (auto mitochondrial antibodies) were detectable over the course of 4 weeks. Furthermore, proteomic analyses showed an enrichment in precursor metabolites for energy and cellular respiration. Investigations after 2h showed a fraction of transplanted mitochondria to be localized within cardiomyocytes.

No immune and inflammatory response nor cytokine activation at 30 days after surgery were also found when a porcine model of ischemia/reperfusion has been used after transplanting autologous pectoralis muscle derived mitochondria [127]. In contrast to the transplantation group, mitochondrial damage and contraction bands could be observed via electron microscopy in the vehicle group. Again, after 4 weeks of recovery no arrhythmogenicity was observed. However, in contrast to the previous report no differences in parameters of global systolic function were found. The majority of mitochondria were present in the myocardium (76.18% ± 11.85) after direct injection in the pig heart. 

Besides the beneficial effects of mitochondrial transplantation directly at reperfusion, even the delayed application 120min after the onset of reperfusion could be shown: in this case, improvements in regional and global myocardial function could be observed.

The group of Guariento et al. addressed the question of the beneficially effect of serial mitochondrial injections on the heart in different settings. In two publications, using autologous pectoralis muscle derived mitochondria from Yorkshire pigs the usage of different protocols (publication 1: single injections 15 min prior to ischemia, 10 injections in 5 min intervals up to 15 min prior to ischemia in alive animals; publication 2: single injection after 20 min warm ischemia and 15 min reperfusion alone compared to an additional second injection 120 min after the start of reperfusion of ex-situ perfused hearts followed by further 2 h reperfusion) was investigated. The authors found beneficial effects of the single injections groups and the serial injections groups compared to the vehicles but no differences between both (single versus serial) [125,126].

An interesting study by Shin et al. investigated multiple aspects of intracoronary mitochondrial transplantation using autologous pectoralis major derived mitochondria from Yorkshire pigs [116]. Besides to positive effects of mitochondrial transplantation on cardiac function, they also had a look on other relevant aspects:

Regarding biodistribution, they showed a specific localization of the mitochondria mostly in the left ventricle. Specifically they found 24.76% ± 2.50 and 23.64% ± 2.42 of mitochondria to be associated with cardiomyocytes and blood vessels, respectively, in their perfused heart model.

Specifically in this study, for two subsets of experiments human cardiac fibroblasts derived with iron (II, III) oxide nanoparticles labeled mitochondria were used for the study of cellular uptake of mitochondria. The human mitochondria were shown to be located in the heart in the interstitial spaces, the vascular walls and within the cardiomyocytes.

In a second subset HeLa and HeLa p0 cell mitochondria were used to investigate the role of mitochondrial viability and respiration competence. Only the application of respiration competent (vital) mitochondria (HeLa) led to an increase in coronary blood flow (CBF).

The application of higher mitochondrial concentrations led to enhanced regional and global left ventricular function but also a significant increase in CBF. Interestingly no change in CBF was seen when the mitochondria were injected directly to the myocardium. The injection of ATP alone led to a similar increase in CBF whereas not so long lasting. The increase in CBF could be replicated by serial mitochondria boli each resulting in an increase of around 100 ml/min. The authors concluded that the increase in CBF might be due to the vasodilatory effect of ATP, which was likely prolonged when respiration competent mitochondria, produced ATP for a longer time period, were applied in contrast to the short term effect of pure ATP application.

The first clinically relevant study was performed using pediatric patients with extracorporeal membrane oxygenation (ECMO) [124]. To our knowledge, this was the first report of a clinical application of mitochondrial auto transplantation. Autologous mitochondria from the rectus abdominis muscle were epicardially injected 2–15 d after ECMO cannulation. 4 of the 5 subjects were successfully separated from ECMO support and showed stable respiratory and renal status (thereby indicating no systemic inflammatory responses). However, 3 of 5 patients survived [124].

Despite being used in the ischemia reperfusion model in the context of cardiovascular research, a recent publication also investigated the effects and the application of mitochondrial transplantation for heart transplantation, thereby widening the possible fields of applications [130]. When gastrocnemius muscle-derived syngeneic mitochondria were injected intracoronary before transplantation and a repeated bolus of mitochondria after transplantation was performed (with 29 h cold ischemia time in between), an improvement of heart function, 20% less necrosis and inflammatory cell infiltration was observed. In a separate set of experiments with Wistar rats, the distribution of the transplanted mitochondria was investigated after intracoronary injection. 10 min after injection, a diffuse distribution throughout the heart was observed [130].

One of the newest applications of autologous mitochondria has been explored by Weixler and colleagues. They used mitochondria for the treatment of right heart failure in a porcine model of pulmonary artery banding and showed beneficial effects compared to untreated animals: Mitochondria-treated animals showed reduced cardiomyocyte loss and a better preserved contractility [133]. Interestingly they also showed in vitro no differences between right ventricular derived and skeletal muscle derived mitochondria as sources for the transplantation experiments.

Although these studies produced positive results with regard to efficacy of mitochondrial transplantation, experiments using mitochondria from diseased patients, which would mirror more closely the real-life situation where this protocol would be applied, are rare. To our knowledge the first study mimicking the transplantation of mitochondria from diseased diabetic animals was conducted by Doulamis et al. in 2020 [134]. In a Langendorff perfused rodent heart model they investigated the transplantation success using either autologous (diabetic) or syngeneic (non-diabetic) mitochondria generated from skeletal muscle tissue. Both groups showed an increase in nearly all myocardial function indices (except LVdevP which was higher when the diabetic mitochondria were used) and a reduction in infarct size without a difference between them. However, the total ATP tissue content was higher when mitochondria from “healthy” non-diabetic rats were transplanted thereby indicating a possible superiority in terms of more advantageous and prolonged effects when using mitochondria from healthy donors. Additionally, in a subset of experiments they showed the successful transplantation of mitochondria from human cardiac fibroblasts into rat hearts via retrograde coronary infusion. In this setting, human mitochondria were shown mostly within myocardial fibers.

Taken together, these studies (almost exclusively conducted by the McCully group) indicate that the mitochondrial transplantation leads to a rescue of myocardial viability and improvements in cardiac functions when applied before or at the start of recirculation. Mitochondria seemingly remained at the place of injection, were incorporated into the target tissue and were viable and present up to 4 weeks after the infarct. Cell culture experiments with iPsS cells derived cardiomyocytes and primary cardiac fibroblasts showed mitochondria to be internalized in the cells via endosomes and lysosomes, progressing the endosomal system, escaping these compartments and being integrated/fusing in the mitochondrial network. Exogenous mitochondria were quite fast present in the cells (after 30 min) near the endogenous mitochondrial network [115]. The incorporated mitochondria were viable, energized and intact. The authors postulated the endogenous mitochondrial proteins (e.g., MFN1, MFN2, OPA1) likely to facilitate the integration into the network.

It has been postulated that the mitochondria, when applied regionally (direct injections or intracoronary) lead to an increase in ATP content of the recipient tissue thereby exerting first beneficial effects and in addition an upregulation of mitochondrial pathway proteins and proteins responsible for energy and cellular regulation precursor metabolites can be seen [128]. Afterwards the mitochondria are then taken up into the myocardium via actin-dependent endocytosis and increase the myocardial cell function by increasing the intracellular ATP content, upregulate proteomic pathways, replace damaged mtDNA, influence cell function and upregulate cardioprotective cytokines. The replacement of damaged mitochondria with respiration competent mitochondria has been shown by Pacak et al. in a transplantation experiment between respiration competent (HeLa) cells and HeLa p0 cells, which have dysfunctional mitochondria [131].

The mechanism how mitochondria migrate to the target cells is still unclear. However, some reports indicate that mitochondria from healthy cells target directly diseased cells with mitochondrial dysfunction [135,136].

Besides the presented promising results, reasonable questions arise, critics are still presented and have to be addressed:

## 7. Safety of Mitochondrial Transplantation

Although most studies report on no or low immunogenic potential of whole intact mitochondria, the immunological reaction against transplanted mitochondria should be properly assessed. In the field of cardiovascular research, various groups reported on the safety of transplanted mitochondria and found no signs of immunological reactions when looking on cytokine markers like hsCRP, IL6 and TNFα, the production of autoimmune responses (auto mitochondrial antibodies) or metabolic changes (respiratory and renal status). One study investigated the immunological reactions in a skin graft model against the single or serial transplantation of syngeneic or allogeneic mitochondria [132]. They found no differences in IL2, IFNγ, IL4, TNFα, IgM alloantibodies, lymphocyte infiltration and survival time of the skin grafts as well as histological changes in heart and lung tissues or liver edema. Especially the investigations regarding skin graft rejection support the statement that whole and viable mitochondria do not lead to an immune response (but rather immunoregulation).

Masuzawa et al. performed extensive investigations using different protocols: As described above, they not only showed no increase of cytokines (IL-1, IL-4, IL-6, IL-12, IL-18, IP-10 and macrophage inflammatory protein-1) or the production of IgM antibodies in vivo, they also showed the successful internalization of autologous liver mitochondria and human- derived mitochondria (Hela cells) within hours into cardiomyocytes in vitro with the preservation of respiratory function. In contrast to an inflammatory response, they found an increase in EGF, GRO, IL-6 and MCP-3, cytokines involved in “angiogenesis, arteriogenesis, immunomodulation, progenitor cell migration, prevention of apoptosis and enhanced salvage and postischemic functional recovery” [128].

In contrast to these promising findings for whole mitochondria, mitochondrial parts or contents (DNA, proteins, lipids) not only seem to have no comparable effects when transplanted but they might even act as inflammatory stimuli [137]. These molecules, also called mitochondrial Damage-Associated Molecular Patterns (DAMPs), which are typically released during the course of cell death, mediate inflammatory immune responses and are associated with pathogenicity and severity of various diseases. Mitochondrial DNA/DAMPs have been found in brain death, trauma patients, sepsis, neurologic (ischemic stroke and bacterial meningitis) and myocardial injury and were associated with early allograft dysfunction and acute respiratory distress syndrome (ARDS) [138,139,140,141,142,143,144,145,146,147,148,149]. McCully et al. compared the transplantation of whole mitochondria, mitochondrial components and frozen-thawed mitochondria [4]. They found a decrease in LVPDP and an increase in LVEDP, a decrease in SS and an increased IR/AAR ratio compared to the transplantation of fresh mitochondria. Furthermore, frozen mitochondria showed decreased oxygen consumption rates compared to freshly isolated mitochondria. In an in vitro setting using HepG2, HeLa, HEK-293 and MCF7 cells as well as primary fibroblast cultures, Kesner et al. found that only intact mitochondria may be incorporated into the cell thereby supporting the previous in vivo findings [150].

In addition, even for whole mitochondria reports on immune responses against the mitochondria exist: Lin et al. found an accumulation of exogenous mitochondria in endothelial cells, which led to an increase in inflammatory cytokines, upregulation of adhesion molecules, alloreactive adhesion and activation of T cells and an increased allograft rejection in a murine model of heterotopic heart transplantation: Coincubation of LMTK-derived mitochondria with mouse endothelial cells led to an upregulation of CD54, CD106 and CD62E as well as MHC II molecules. Mitochondria treated endothelial cells had a higher adherence of allospecific T cells. Coincubation of Human aortic endothelial cells (HAECs) with mitochondria from HeLa cells resulted in an upregulation of CD54, CD106 and CD62E and an increased production of IL-6, IL-8 and MCP-1 [151]. Coincubation of murine mitochondria with dendritic cells resulted in an up regulation of CD40 and CD86 as well as MHC II. Additionally co-incubation of human PBMCs with mitochondria treated ECs resulted in increased numbers of effector (IFNγ+, TNFα+) CD8+ T cells [151]. Also, other studies report on inflammatory responses to mitochondria, for example, when activated platelet-derived mitochondria where given intravenously causing transferation to neutrophils and cytokine production or when lymphoblast derived mitochondria led to the modulation of cytokine production in macrophages and dendritic cells [152]. Boudreau and coworkers showed that the release of free and encapsulated mitochondria by platelets led to inflammatory reactions [153]. These findings are supported by higher levels of free mitochondria in the course of different pathological conditions [153]. However it seems to be unclear if these diseases possibly accompanied by cell death and mitochondria releases lead to inflammatory responses or if the inflammatory reaction appears in the course of the disease without the stimulating effect of released mitochondria or mitochondrial components. 

## 8. Optimization of Mitochondrial Delivery 

Although mitochondrial transplantation has been proven successful in a variety of different models, species and settings, the potential for optimization of this therapeutic approach regarding the application route, improvements of the effect strengths and duration with adapted delivery systems or repeated injections is still given.

Despite controversial reports of the safety of mitochondrial transfer and transplantation, various approaches have been used to optimize mitochondrial applications. Centrifugation of cells has been shown to increase the mitochondrial transfer efficiency between them; magnetomito transfer, EGF, Pep-1, transactivator of transcription (TAT) peptide, triphenylphosphonium cation (TPP) nanocarrier, MitoCeption, mitochondrial transformation or TAT-dextran-modified mitochondria (TAT-Mito) are other possibilities and currently investigated [110,117,150,154,155]. Even the direct targeting of the mitochondria, for example, via alterations using liposome-based carriers (MITO-Porter) offers interesting options for future therapeutic approaches/modifications [156]. Another, more biologically based approach was investigated in cell culture conditions by Phinney et al.: They showed that when MSCs and macrophages were co cultured, MSCs produce arrestin domain-containing protein 1-mediated MVs (ARMMs) to exclude mitochondria, which are then integrated into macrophages. Interestingly, the MSCs were also shown to excrete exosomes including microRNAs which exhibit regulatory functions, tolerizing the macrophages by modulating inflammatory signaling pathways especially Toll-like receptor (TLR) expression [111]. Mahrouf-Yorgov et al. described a physiological mechanism leading to the mitochondria biogenesis/export: cocultivation of MSCs with damaged cells seemed to activate rescue properties due to the sensing of DAMPs which resulted in an engulfment of mitochondria by MSCs and promoting mitochondrial biogenesis [157]. However, if these generated mitochondria produced as a consequence of sensing DAMPs induce beneficial effects in vivo is in question. The possibility that other molecules might be also secreted, which would induce inflammatory responses in vivo has to be excluded prior to further applications. Another study proved this principle by co culturing human MSCs with human monocyte–derived LPS stimulated macrophages also showing the excretion of extracellular vesicles which were taken up by the macrophages dampening the proinflammatory responses, promoting phagocytic capacity and an anti-inflammatory phenotype [112].

The applied protocols have been proven to produce viable and respiration competent mitochondria with maintained membrane potential. However, whereas early protocols took up to 90min for mitochondria generation making them time consuming and critical for application in patients needing immediate treatment, new optimized protocols could significantly reduce this time so that mitochondria generation can nowadays be achieved in less than 30min [114]. Furthermore, regarding long term effects follow up was performed for up to 4 weeks [127,128] and the repeated dosing has been also investigated implicating no superior effect of serial mitochondrial injections. In general, the beneficial effect of transplanted mitochondria seems to be not directly dose dependent as evidenced by various studies. In some of the studies a transient increase of coronary blood flow over 15min [122] and increasing with escalating mitochondria dosages has been observed. This increase might be attributable to the increase in ATP content delivered by the application of mitochondria and had no visible effects on cardiac functions. The beneficial effects of mitochondrial transplantations appeared often with lower than maximal dosages. In contrast, when used in a heart transplantation setting, higher dosages than previously used were necessary to induce a positive effect. The authors attributed this to the higher metabolic rate of their murine animal model [130].

## 9. Storage Of Mitochondria

Using the promising effects of transplanted mitochondria without the necessity to take autologous muscle biopsies and preparing them for transplantation in a time-pressing setting would be tempting. So the consideration to preserve freshly generated mitochondria for later use seems obvious. The issue of using fresh vs. frozen mitochondria and the usage of mitochondrial particles of DNA/RNA has been investigated/addressed by McCully et al. in 2009 [4]. Whereas freshly transplanted mitochondria resulted in a recovery of cardiac function, frozen mitochondria, mitochondrial components, ATP or DNA/RNA resulted in a decrease of function. Compared to fresh used mitochondria, frozen mitochondria showed a decrease in oxygen consumption and bioenergetics of up to 90% [4]. Effects of the sole application of ATP, which seems to induce one of the first beneficial effects after transplantation are questionable due to its short duration time [4,116].

Using various cryopreservation protocols, different groups showed preserved mitochondrial functions and no observable histological damages from brain tissue as well as cardiac and skeletal muscles [158,159,160,161]. The further optimization of conservation protocols might enable the long term storage of mitochondria for use on demand.

## 10. Using Stem Cells for Mitochondrial Transplantation

Mitochondria for in vivo cardiovascular applications are usually derived from autologous muscle biopsies and produce significant therapeutic effects. But due to demographic changes and the increased occurrence of cardiovascular diseases the patients relevant for these therapeutic strategies are usually older than the currently used (young) animal models in which these strategies have been tested. In addition inherited mitochondrial diseases would prohibit the usage of autologous mitochondria. This poses the question if the traditionally used muscle biopsies are optimal for this approach and if not other cell sources or strategies could be options and used as donors for the applied mitochondria.

Although autologous derived mitochondria from the skeletal muscle are currently the most common procedure for mitochondrial transplantation in the field of cardiovascular research, other sources of mitochondria have also been successful used: Transplantation of autologous mitochondria from heart tissues and liver tissues as well as allogeneic and syngeneic derived mitochondria have been described. Especially in the field of neuroscience, stem cells are common sources. The principal proof that mitochondria can be transferred into homogeneic and xenogeneic cells has been shown by various groups: Kitani et al. co-incubated these cells successfully with isolated mitochondria [162]. Stem cell derived mitochondria have been used for transplantation in cancer cells [154]. Islam et al. showed the successful mitochondrial transfer from human and mouse BMSCs to mice alveolar epithelium by forming gap junction channels and mitochondria-containing microvesicles leading to the presence of human mtDNA and an increase of ATP content in the alveolar tissue. When HeLa cell-derived mitochondria were used for transplantation in rabbit cardiomyocytes a co localization at the cell surface within 2h followed by an internalization in the myocytes within 8h was seen. However, in this study, the majority of mitochondria remained in the extracellular space and just 3 - 7% of mitochondria seemed to be incorporated [163].

The fact that mitochondria provoke lower or no immunogenicity compared to cell-based approaches and that they are significantly smaller thereby reducing the risk for cell-induced vessel occlusion, transplantation options from different individuals or species may be worth investigating [164].

Interestingly, an increased mitochondrial transfer efficacy was observed for iPS-derived MSCs than for BM MSCs when MSCs were co cultured with rat airway epithelial cells and in vivo [165]. In a mouse model of anthracycline-induced cardiomyopathy iPSC-MSCs displayed a higher human mitochondrial retention rate compared to BM-MSCs [166]. This might be due to a high expression of MIRO1 and TNFαIP2 which made iPSC-MSCs “more responsive to TNFα-induced tunneling nanotube (TNT) formation for mitochondrial transfer to CMs, which is regulated via the TNFα/NFκB/TNFαIP2 signaling pathway” [166].

Using healthy non-autologous mitochondria might even be more beneficial than using autologous mitochondria. For example mitochondria in diabetic patients and rodent models have been shown to be dysfunctional, having reduced respiratory capacity and ATP production rate [134]. In addition, autologous mitochondria might not be an option in patients with mitochondrial myopathies and mutational changes in the mtDNA [167].

Although already applied (often investigated in subgroups of the experimental studies; see section “Mitochondrial transplantation for heart diseases”), the usage of stem cell derived mitochondria for in vivo purposes is currently under-investigated and important questions for practical considerations, like identifying the best cell source, isolation protocols, improvements of mitochondria quantities and preservation of these mitochondria are worth to be further examined. Traditionally HSCs were thought to be mitochondria sparse but recent studies report on abundant mitochondria: Almeida showed a high mitochondrial mass and high mtDNA content in HSCs. mtDNA content declined with differentiation of the HSCs. But baseline oxygen consumption, mitochondrial ATP production and maximal respiratory capacity were lower in HSCs compared to MMPs and CPs; turn over capacity of mitochondria was low in HSCs.

The importance of identifying the best cell source has been recently evidenced by an investigation from Paliwal and colleagues. In their study SCs from dental pulp or Wharton jelly showed better efficacy for transfer than other stem cells. The authors concluded that a careful selection on cell source should be made since MSCs with higher mitochondrial bioenergetics display higher rescue potential with lower mitochondrial transfer [168].

Another aspect which should be regarded is the fact, that mitochondria or better said the cell type and tissue differs in metabolic profile, mitochondrial content and energy sources and demands using glucose, fatty acids, ketone bodies or lactate as source. In the healthy heart, mainly (>95%) fatty oxide oxidation occurs, which switches to glycolytic pathways in the failing heart [169]. A direct comparison between brain, heart, liver, skeletal muscle and kidney of Wistar rats revealed hearts having a 5–10 fold higher cytochrome C oxidase activity than the other organs with liver being the lowest. However, the CoX/CS ratio was the highest in liver and kidney. mtDNA and mitochondrial content per cell were the highest in cardiac muscle, followed by skeletal muscle [170]. However, in vivo studies using different mitochondrial sources seemed to display no functional differences [128], which was confirmed by investigations of different muscle sources for in vitro mitochondrial transfer into cardiomyocytes [133].

The subpopulations of mitochondria, SSM vs IMF, have been shown to differ in regard of morphology and functionality [171]. These two subpopulations seem to exhibit different age-related metabolic alterations: whereas SSMs are mainly unaltered IMF exhibit alterations in oxidative phosphorylation [172]. However, no functional differences were observed in transplantation experiments [4]. Studies indicate however different susceptibilities of mitochondrial subtypes to diseases, which should be taken into consideration when treatments for affected patients are considered.

## 11. Possible Approaches:

When using mitochondria for future transplantations different scenarios seem to be possible:

Rejuvenating of mitochondria from old stem cells (e.g., reprogramming, miRNA treatments or preconditioning modifications) or the use of non-autologous stem cell mitochondria either from healthy donors or from cell cultures are interesting options. However, the effect of rejuvenation strategies is still under investigation and not always successful: using transplantation of stem cells from old to young mice for rejuvenation of these cells showed no significant effects regarding the generation of downstream progenitors myeloid-biased differentiation in one study [173]. Further developments in genetic and pharmacological methods might increase the mitochondrial potential of autologous derived mitochondria. Especially a combination of methods, for example, by using miRNAs might potentiate the beneficial effects of mitochondria.

The recent identification of tissue heterogeneity, cell heterogeneity and even intra cell heterogeneity of mitochondria holds great promise for application in mitochondrial transfer/transplantation. Identifying the most promising meaning least senescent stem cells and mitochondria could aid in their application.

From a metabolic point of view the stem cell origin should not be so important: Despite being from another energetically background, the line of evidence suggests that mitochondria from different origin (in vivo liver and different skeletal muscles; in vitro stem cells) are able to be incorporated into target cells and to exert positive effects in heart muscle. From a genomic point of view, mitochondria from cells that underwent only a limited number of divisions but are not quiescent might be a good source. Mitochondria from somatic tissues seem to harbor the risk of accumulated DNA damage which might impair their reparative effect. The influence of impaired fission and fusion of old mitochondria might influence regenerative capacity of transplanted mitochondria from old donors. However, on the positive site for autologous mitochondria stands that they are readily available within 30min and that they are immunogenic safe. However, in vivo studies showed no immunogenic potential of intact respiring mitochondria and when external sources are used, they could be available even faster than autologous freshly derived mitochondria.

The obvious age effects of autologous (muscle) derived mitochondria and additionally possible underlying disease effects based on mitochondrial or metabolically impairment might worthen investigating other mitochondria sources. As shown stem cells pose promising candidates for exerting even more beneficial effects.

Optimizing the application process, for example, identifying the most promising tissue and stem cell source, the generation of mitochondria with reduced immunogenicity and developing conservation methods for the readily applicable usage of the generated mitochondria might enhance and promote this interesting therapeutic field. By applying screening methods for mitochondria to be used, possible underlying mtDNA defects might be prevented. In this context, recently a new method for mtDNA sequencing (STAMP) has been proposed which could aid in identifying the optimal mitochondria for transplantation [174].

## 12. Conclusions

Despite previous promising usage of mitochondria for transplantations important considerations regarding significant aging effects of somatic cells, stem cells and mitochondria as well as factors like safety issues, tissue sources and possible disease effects deserve further investigations when mitochondrial transplantations are to be used for future applications. Factors influencing stem cell and mitochondria function include age of the cells, probably previous divisions of the cells, heterogeneity of stem cells as well as mitochondria and likely tissue source and additional diseases. Furthermore after the first positive reports, the time of treatment for the most beneficial effect and repetitions of applications should be further investigated: positive effects have been shown pre ischemia, prior to and during reperfusion as well as after delayed application. The quantity of mitochondria seems to be less critical as only a small number of mitochondria is needed for improving cardiac functions. The development of further safety and storage solutions for mitochondria could improve applications. Following the first promising reports of stem cell derived mitochondria further research especially considering the differences of autologous (maybe collection in early life stages and asservation for later use) vs. allogeneic vs. syngeneic sources deserve further investigations and will surely lead to exiting new developments during the upcoming years.

## Figures and Tables

**Table 1:** Overview over cardiovascular mitochondrial transplantation studies.

Study	Model	Mitochondrial Source	Protocol/Application	Outcomes
Doulamis 2020 [134]	Diabetic and non-diabetic rats	-autologous or from diseased animals -pectoralis major muscle	-ischemia/reperfusion in Langendorff perfused hearts in a T2D model -antegrade i.c.	-Prior: mitochondrial ATP content ↓ in diabetic rats -LVdevP ↑ in both groups (higher in group with healthy mitochondria), LVEDP ↓ + IS ↓in both groups →no difference in cardiac function, IS and CBF between both groups (except LVdevP) -tissue ATP ↑ (higher in group with healthy mitochondria)
	rats	-syngeneic -pectoralis major muscle	-for biodistribution -antegrade i.c.	-distributed throughout the heart
		-human cardiac fibroblasts	-for biodistribution -retrograde i.c.	-distributed within myocardial fibers
Ramirez-Barbieri 2019 [132]	mice	-syngeneic + allogeneic -gastrocnemius + quadriceps femoris muscle	-single or serial i.p. in a skin graft model	-no inflammatory or autoimmune responses (IgM antibodies, IL4, TNF-α, skin graft survival time, lymphocyte infiltration, free mtDNA, histological changes in heart and lung)
Moskowitzova 2019 [130]	mice	-syngeneic -gastrocnemius muscle	-heart transplantation -29h cold ischemia time -i.c. before transplantation + 5 min after transplantation	-beating score ↑, EF ↑and FS ↑, necrosis and inflammatory cell infiltration ↓ -no differences in TUNEL^+^ nuclei -myocardial injury (contraction bands) in vehicle groups
	rats		-antegrade i.c.	-diffuse distribution in the heart
Guariento 2020 [125]	pigs	-autologous -pectoralis muscle	-circulatory death/Ischemia/cardioplegia/reperfusion -single or two injections (2^nd^ after 2 h of ex-situ heart perfusion and reperfusion for additional 2 h)	-LV/ventricular peak developed pressure ↑, FS ↑, myocardial oxygen consumption ↑, IS ↓
Guariento 2020 [126]	pigs	-autologous --pectoralis major muscle	-preischemic intracoronary single or serially injections -ischemia/reperfusion	-CBF ↑ pre-RI period and during reperfusion -myocardial function ↑ (EF ↑ + LV Pdev ↑, LV dP/dt max↑, LV Ped ↓ SS ↑, FS ↑), IS ↓; necrosis + edema ↓, preserved mitochondrial structure → no differences between single and serial injections
McCully 2009 [129]	rabbits	-allogenic -left ventricle	-ischemia/reperfusion-in excised hearts	-LVPDP + SS + ATP content ↑, LVEDP + IS + CK-MB and cTnI, caspase-3-like activity ↓, -no differences in TUNEL^+^ nuclei -mitochondria viable and detectable; located near the myocytes
			-frozen mitochondria/ mitochondrial components or mitochondrial DNA/RNA	-LVPDP ↓, LVEDP ↑, SS ↓ + IS ↑, RCI ↓, oxygen consumption ↓ compared to fresh mitochondria
Masuzawa 2013 [128]	in vivo: rabbits	-autologous -pectoralis major muscle optical mapping: liver mitochondria	-ischemia/reperfusion recovery for 2h or 28days	-no arrhythmogenicity, decreased necrosis, enhanced postischemic function, IS ↓, CK-MB ↓ and cTnI ↓, increase of tissue ATP content in AAR, percent systolic wall thickening ↑ compared to vehicle, segmental hypokinesis in vehicles TUNEL^+^ nuclei ↓ and caspase 3 activity ↓, no inflammatory effect (hsCRP↓, IL6↓, TNFa↓), no antimitochondrial antibodies after 4 weeks -proteomic analyses: generation of precursor metabolites for energy ↑ and cellular respiration↑ -mitochondria in the interstitial spaces surrounding cardiomyocytes at 0, 2, 4, 8 and 24 h following injection-fraction of the labeled mitochondria within cardiomyocytes 2 h after injection
		-xenogeneic human mitochondria from HeLa cells	separate group: -biodistribution -injection in tissue	-majority of mitochondria in the interstitial spaces, partly localized within cardiomyocytes
	in vitro	- HeLa- derived intact and sonicated mitochondria	subgroup: -co culture of HeLa- derived intact and sonicated mitochondria and human PBMCs	no upregulation of cytokines associated with rejection (IL-1, IL-4, IL-6, IL-12, IL-18, IP-10 and macrophage inflammatory protein-1a and -1b) EGF↑, GRO↑, IL-6↑ and MCP-3↑ →associated with enhanced postinfarct cardiac function
	in vitro	-syngeneic -rat liver mitochondria	subgroup: -internalization -neonatal rat cardiomyocytes cocultured with mitochondria	-majority of mitochondria internalized within 24 h of incubation -oxygen consumption ↑
	in vitro	-xenogeneic -HeLa cell mitochondria	subgroup: -internalization -cardiomyocytes incubated with mitochondria	-mitochondria adherent to cells within 2 h and internalized within 8–24 h -no colocalization with lysosomes or autophagosomes
Kaza 2017 [127]	pigs	-autologous -pectoralis major muscle	-ischemia/reperfusion -subendocardial injections -4 weeks recovery	-IS↓, no immune and inflammatory response and cytokine activation, no arrhythmia, -mitochondrial damage and contraction bands in vehicle hearts -no differences in systolic function (EF, FS and global circumferential strain)
Blitzer 2020 [122]	pigs	-autologous -pectoralis major muscle	-ischemia/reperfusion -delayed i.c. application	-LVEF↑, EF ↑, FS ↑, fractional area change, IS ↓, LV EDP ↓ -maximal rate of rise of LV pressure ↑, SS↑, LVFS↑, LVFAC↑, transient CBF ↑
Emani 2017 [124]	children	-autologous -rectus abdominis muscle	-epicardial injection 2–15 d after ECMO cannulation	-4/5 improvement in ventricular function separation from ECMO, 3/5 survived -no signs of immune responses (respiratory and renal status)
Shin 2019 [116]	pigs	-autologous -pectoralis major muscle	safety and biodistribution: -i.c. serially autologous injections, increasing concentrations subgroup: -efficacy in ischemia/reperfusion	-mitochondria located in the LV, partly in the arterial sheath and in the right carotid artery, small amount in descending aorta -mitochondria within cardiomyocytes, interstitial spaces and the vascular walls - enhanced regional and global LV function; CBF ↑ in higher concentrations; CBF↑ i.c., no increase after injection -Improved myocardial function (FS↑, proportion LV fractional area change, EF ↑), IS↓
			-devitalized mitochondria + mitochondria from HeLa- and HeLa-p0 cells	-HeLa mitochondria: CBF ↑ CBF ↑ after i.c. injection of ATP but shorter duration
Weixler 2020 [133]	in vitro	-autologous -heart muscle, soleus muscle, gastrocnemius muscle	-internalization -neonatal rat RV cardiomyocytes	-no differences in ATP content or mitochondrial internalization
	in vivo: pigs	-autologous -gastrocnemius muscle	-injection into RV free wall after pulmonary artery banding	-TAPSE ↑, contractile function↑, apoptosis ↓, myocardial fibrosis↓
Cowan 2016 [123]	rabbits	-human adult cardiac fibroblasts	-imaging -Langendorff hearts -global and regional Ischemia/reperfusion -i.c. or local injection	-most of mitochondria remained within heart throughout reperfusion -majority within interstitial spaces between cardiomyocytes, partly co localization with cardiomyocytes
		-autologous -liver mitochondria	function: -no ischemia or regional ischemia/reperfusion -antegrade i.c.	myocardial function ↑ (EDP↓, IS ↓, SS ↑ and dP/dt ↑)

## Abbreviations

AAR	area at risk
BM	bone marrow
CBF	coronary blood flow
CDC	cardiosphere-derived
CPC	cardiac progenitor cells
DAMP	damage-associated molecular pattern
dP/dt	derivative of pressure
ECMO	extracorporeal membrane oxygenation
EV	extracellular vesicles
FS	fractional shortening
HSC	hematopoietic stem cells
IMF	intermyofibrillar mitochondria
IS	infarct size
LV	left ventricle/left ventricular
LVdevP	left ventricular developed pressure
LVEDP	left ventricular end diastolic pressure
LVFAC	left ventricular area change
LVPDP	left ventricular peak developed pressure
MPTP	mitochondria permeability transition pore
MN	mononuclear
MSC	mesenchymal stem cells
mtDNA	mitochondrial DNA
RCI	respiratory control index
RI	regional ischemia
ROS	reactive oxygen species
SC	stem cells
SS	systolic/segmental shortening
SSM	sub sarcolemmal mitochondria
TAPSE	Tricuspid annular plane systolic excursion
T1D	type 1 diabetes
T2D	type 2 diabetes
